# Integrating Care Coordination, Education, and Research in Mobile Health and Wellness Programs

**DOI:** 10.1093/geroni/igaf122.272

**Published:** 2025-12-31

**Authors:** Lana Sargent, Patricia Slattum, Kimberly Battle, Martha Purvis, Katherine Falls, Amy Salisbury, Jack Weisskohl, Faika Zanjani

**Affiliations:** Virginia Commonwealth University School of Nursing, Richmond, Virginia, United States; Virginia Commonwealth University College of Health Professions, Richmond, Virginia, United States; Virginia Commonwealth University, Richmond, Virginia, United States; Virginia Commonwealth University School of Nursing, Richmond, Virginia, United States; Virginia Commonwealth University, Richmond, Virginia, United States; Virginia Commonwealth University School of Nursing, Richmond, Virginia, United States; Virginia Commonwealth University School of Nursing, Richmond, Virginia, United States; Virginia Commonwealth University, Richmond, Virginia, United States

## Abstract

The VCU School of Nursing’s Mobile Health and Wellness Program (MHWP) is an age-friendly interdisciplinary mobile clinic operating in rural and urban Virginia. MHWP serves three critical purposes for the Commonwealth of Virginia and VCU: First, MHWP decreases barriers for medically under-resourced populations by bringing care and coordination of services directly to communities. Second, MHWP increases the number of interdisciplinary healthcare professionals prepared to meet the needs of medically under-resourced communities through interdisciplinary clinical learning experiences led by VCU faculty. MHWP facilitates the training of a public-ready healthcare professional workforce. Third, we integrate transdisciplinary research. We operate in nine under-resourced areas in Virginia, including six urban low-income housing areas and two rural sites. Decreasing barriers to care through care coordination resulted in an 8.6% reduction in emergency room visits and a 9.8% reduction in hospitalizations. HRSA funding to develop a nurse-led, interprofessional mobile health clinic through which we broadened the program’s reach to two additional urban sites and two distant rural communities, further increasing the program’s scope to become the VCU Mobile Health and Wellness Program. In 2022, we broadened our intentional community engagement, education, and research efforts to include four additional communities. We purchased two clinical vehicles to reach these sites and provide care. MHWP is an example of how a flexible approach works best when teams are prepared to meet specific communities’ social, economic, and health needs within cultural and linguistic characteristics.

